# Supplementary material for: Characterization of Ultrafine Particles and VOCs Emitted from a 3D Printer
Source: Int J Environ Res Public Health. 2021 Jan 21;18(3):929. doi: 10.3390/ijerph18030929 (PMC7908560; doi:10.3390/ijerph18030929)
Supplement: Supplementary file 1 [file ijerph-18-00929-s001.pdf]

Supplementary Materials

1. Results of Particle Number Concentration and Particle Size Distribution

The average and maximum values of size differentiated concentrations (determined by SMPS), the total number of concentrations (using CPC), and their standard deviations for the individual filaments are shown in Table S1.

Table S1. Particle number concentration of all measured samples.

Measurements	Background PNC (SD) (#/cm³)	Heating PNC (SD) (#/cm³)	Printing PNC (SD) (#/cm³)	Printing Peak (#/cm³)	Printing Mode (nm)	Cooling PNC (SD) (#/cm³)
Zero-test	5,337 (161)	5,159 (65)	4,692 (310)	5,589	115.5	4,113 (97)
PETG	4,086 (207)	5,357 (2030)	86,422 (27,757)	131,020	86.4	49,585 (5,508)
NGEN	2,604 (124)	2,507 (120)	16,722 (8,894)	37,276	115.5	11,176 (1,097)

PNC: particle number count = total number concentration; SD: standard deviation.

The following graphs present (Figure S1–S3) the SMPS output, that is the size distribution of the aerosol over time, i.e., the distribution of concentrations from the start to the cooling phase of the printing. The normalized particle concentration dN/dlogDp is plotted on the vertical axis (concentration is weighted by the width of the channel interval; dN is the number of particles, Dp is the mean diameter of the interval) in relation to the unit of air volume (cm^3). The horizontal axis shows the aerodynamic diameter of the particle in nm and the measurement of time in minutes. In the graphs of Figures S1 and S2, it is possible to observe an increase in concentrations of particles with a median of 20 nm and 11.5 nm mode in the first minutes of the start of the printing of PETG, or a median of 50 nm and 48.7 nm mode at the start of the printing of NGEN. Then the median shifts over time toward larger particles due to their coagulation. These results confirm the development of particles smaller than 100 nm in the printing of both filaments. In variants of the b) graphs in Figures S1 and S2, it can be observed that the most abundant size of the particle number concentration in the sampling reaches values of 6.10^5 #/cm³ during the printing of PETG, or 7.10^4 #/cm³ during the printing of NGEN.

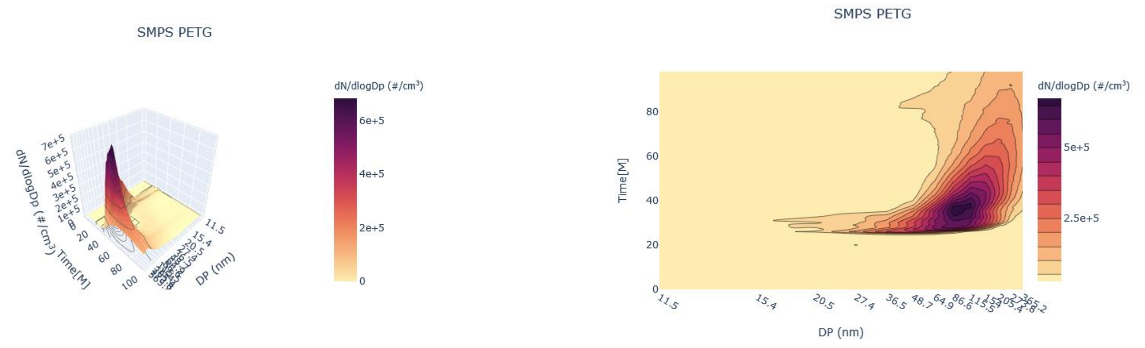

(**a**) (**b**)

Figure S1. Particle size distribution before, during and after printing with PETG filament (**a**) view of data from the end of the measurement during cooling; (**b**) a detailed view of the data from the beginning of the measurement.

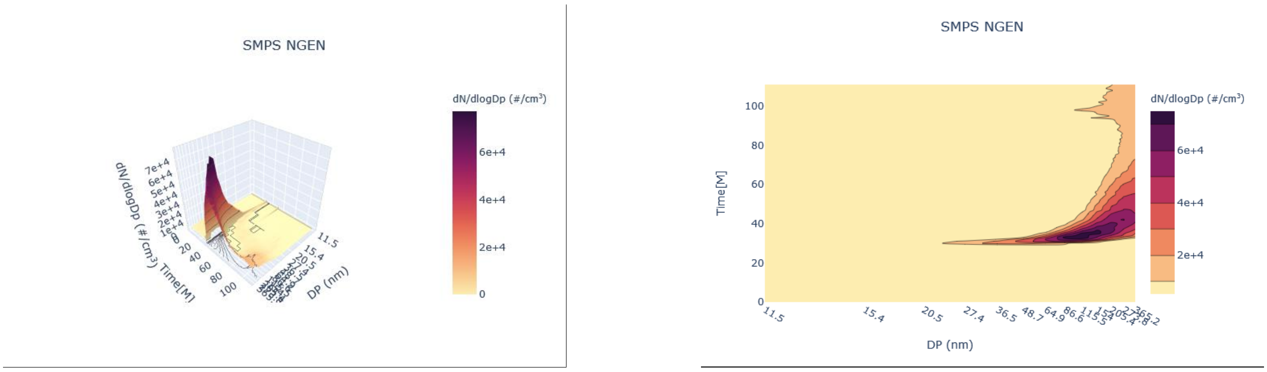

(**a**) (**b**)

Figure S2. Particle size distribution before, during and after printing with NGEN filament (**a**) view of data from the end of the measurement during cooling; (**b**) a detailed view of the data from the beginning of the measurement.

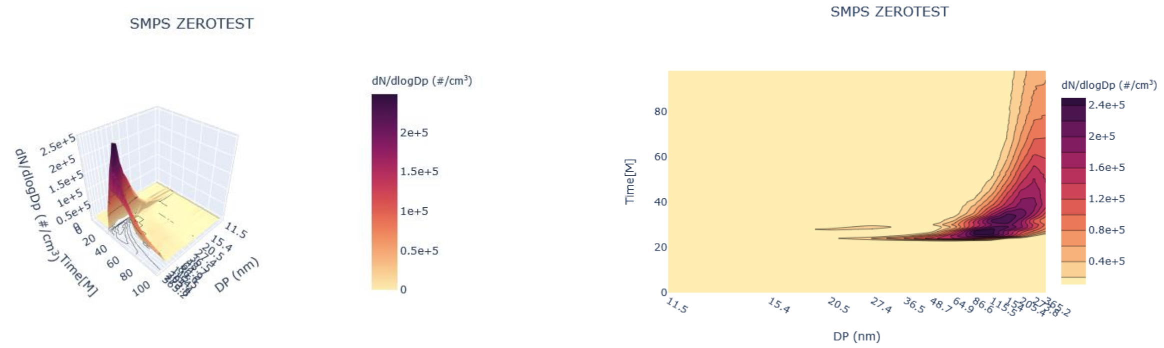

(**a**) (**b**)

Figure S3. Distribution in filamentless printing, so-called Zero-test (**a**) view of the data from the beginning when measuring the background; (**b**) view of the data from the end of the measurement during cooling.

2. Results of VOC Qualitative Analysis

The results of the qualitative analysis of 3D printing with the PETG filament at extruder temperatures between 220 °C and 270 °C showing the relative concentrations expressed by the increase in area corresponding to Quant Ion on TIC during the 3D printing are shown in Figure S4. The dominant compounds at all printing temperatures are again Xylene, Toluene and Ethylbenzene. Present at all temperatures are 1-Octanol, Trimethylbenzene, Nonanal, Napthalene and Decanal. Benzene appears at the extruder temperature of 250 °C. Benzene was also identified at the extruder temperature of 220 °C, but the relative value of its Quant Ion Intensity is very low, 8.4 times lower than at the extruder temperature of 270 °C (see Figure S5).

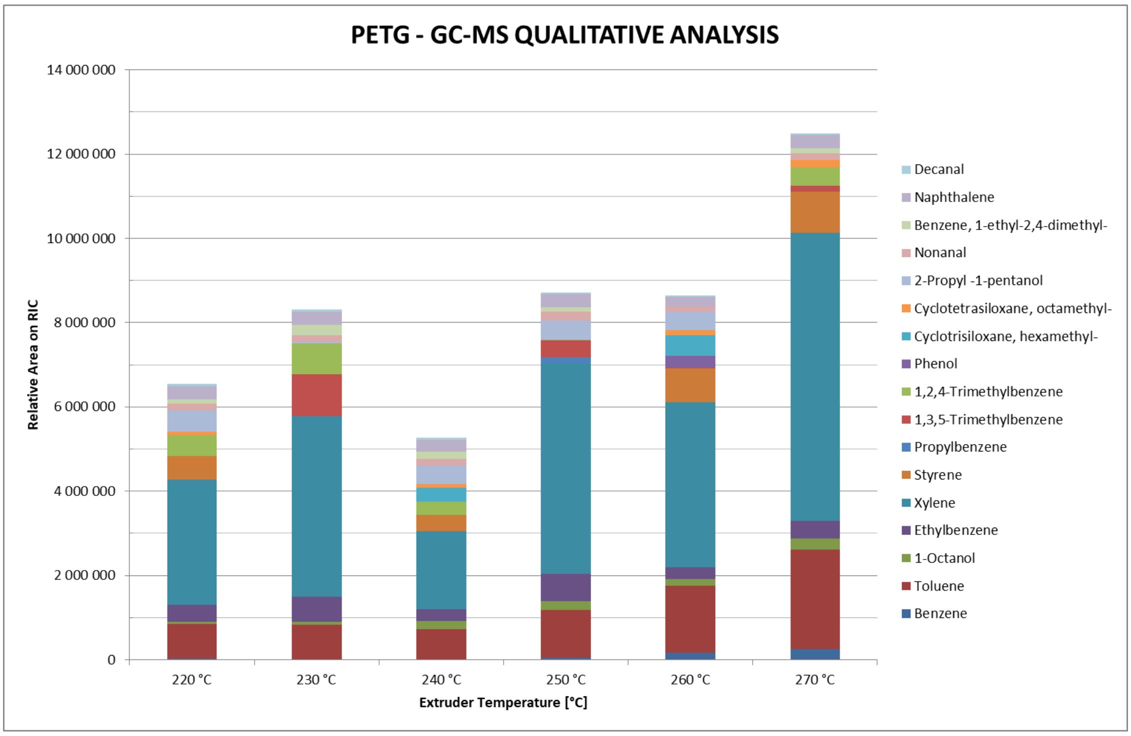

Figure S4. Qualitative GC-MS analysis –PETG filament.

To allow mutual comparability, Figure S5 shows the Quant Ion Intensity for the individual compounds identified in the qualitative analysis after the completion of PETG filament 3D printing at extruder temperatures between 220 °C and 270 °C.

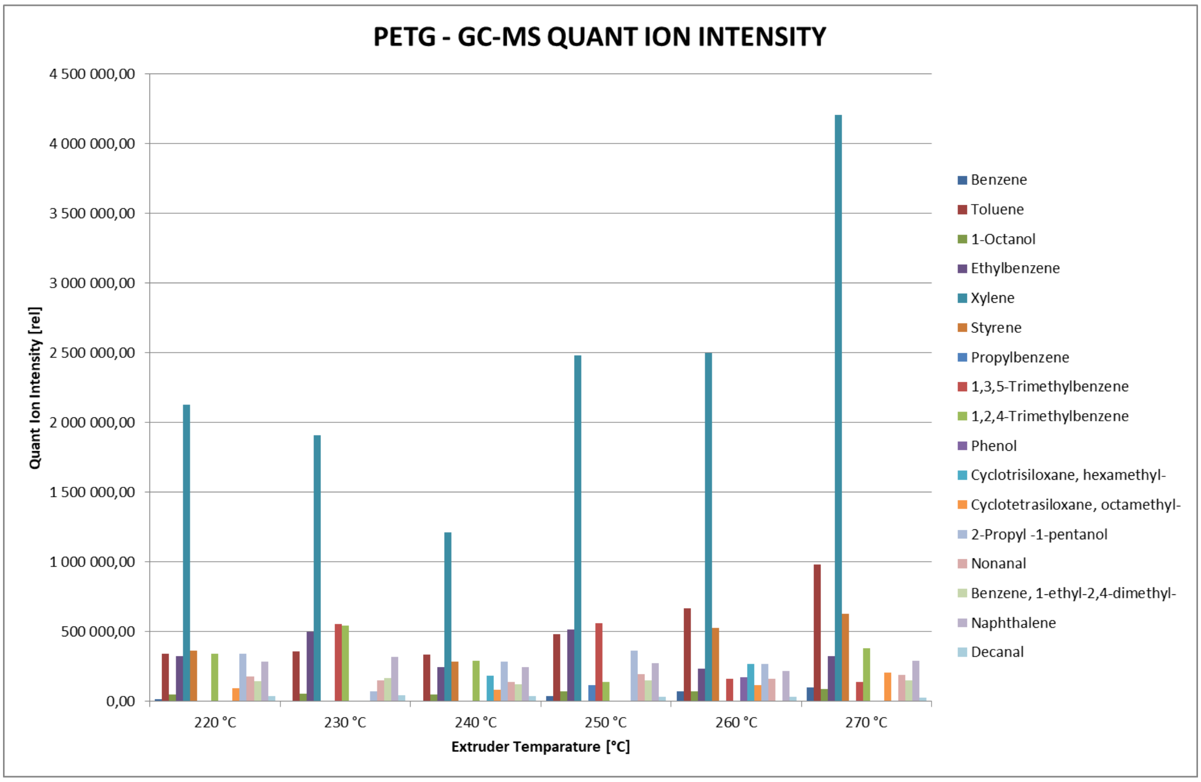

Figure S5. Quant Ion Intensity – PETG filament.

The results of a qualitative analysis of 3D printing with the NGEN filament for extruder temperatures between 220 °C and 270 °C showing the relative concentrations expressed by the increase

in area corresponding to Quant Ion on the TIC during 3D printing are shown in Figure S6. The dominant compounds present at all printing temperatures are Xylene, Toluene and Ethylbenzene. Nonanal, Napthalene, Decanal, Benzene 1-ethyl-2.4-dimethyl- and Trimethylbenzene are present at all temperatures. The presence of Benzene appears starting from the extruder temperature of 250 °C, the relative value of its Quant Ion Intensity rises with the increasing extruder temperature (see Figure S7).

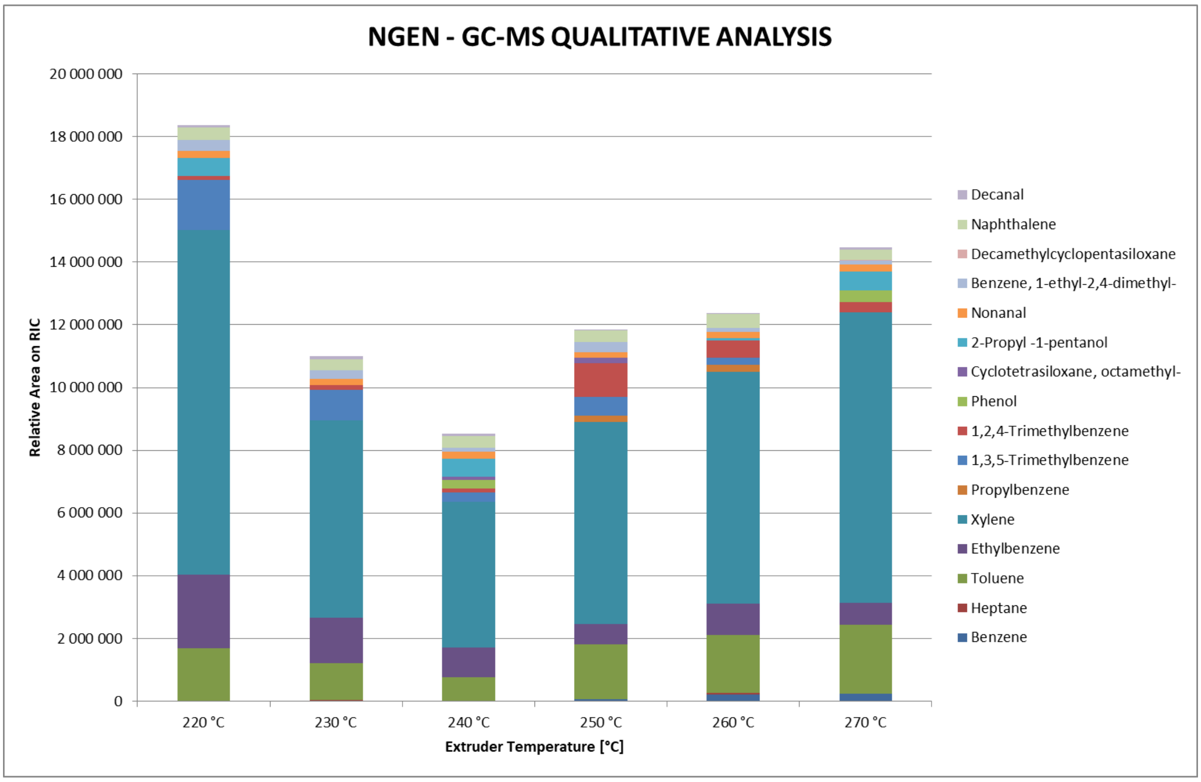

Figure S6. Qualitative GC-MS analysis –NGEN filament.

To allow comparability, Figure S7 shows the Quant Ion Intensity for the individual compounds identified in the qualitative analysis after the end of the 3D printing with the NGEN filament at extruder temperatures between 220 °C and 270 °C.

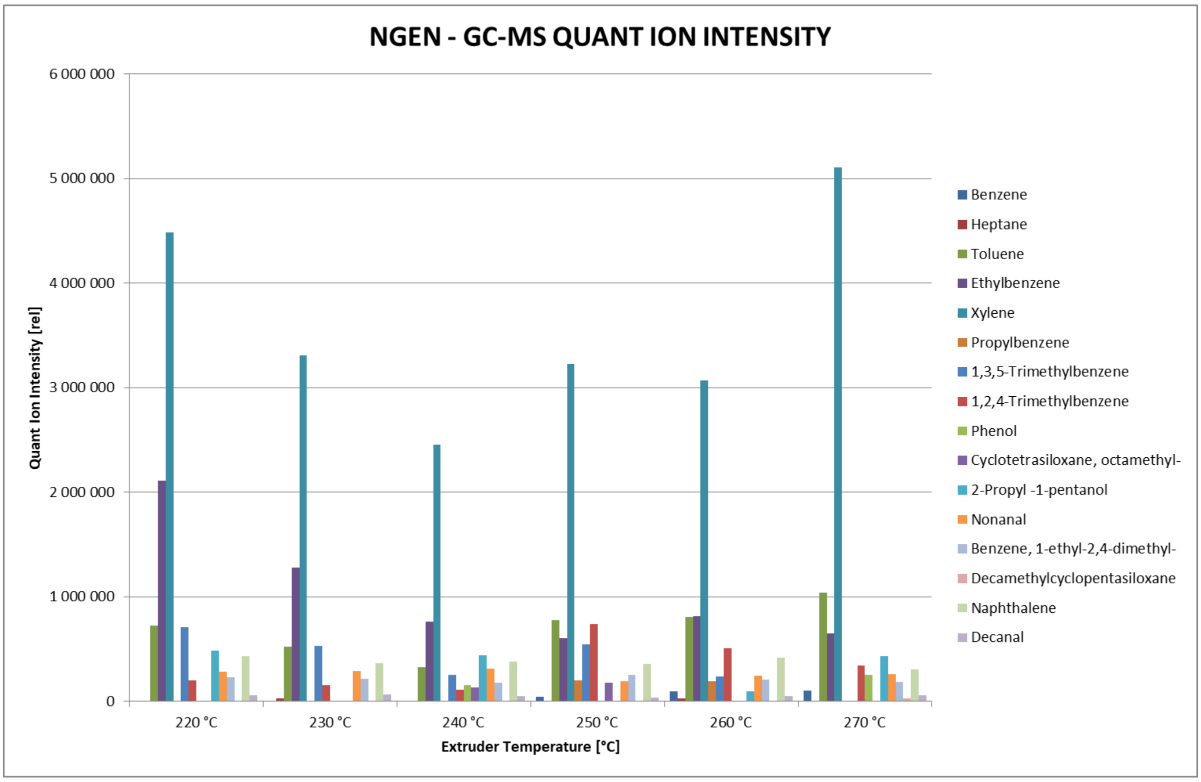

Figure S7. Quant Ion Intensity – NGEN filament.
